# Impact of value-based care on quality of life, clinical outcomes, patient satisfaction, and enhanced financial protection among hypertensive patients in Ghana: A protocol for a mixed method evaluation, 2024

**DOI:** 10.1371/journal.pone.0320861

**Published:** 2025-04-01

**Authors:** Duah Dwomoh, Gifty Sunkwa-Mills, Kwasi Owusu Antwi, Maxwell Akwasi Antwi, Tobias Floris Rinke de Wit

**Affiliations:** 1 School of Public Health, College of Health Sciences, University of Ghana, Accra, Ghana; 2 PharmAccess, Accra, Ghana; 3 PharmAccess, Amsterdam, the Netherlands; 4 Department of Global Health, Amsterdam Institute for Global Health and Development, University of Amsterdam, Amsterdam, The Netherlands; World Health Organization, Regional Office for South-East Asia, INDIA

## Abstract

**Background:**

Evidence on which hypertensive intervention is cost-effective is essential to inform strategy, policy development, practice, implementation, and resource allocation. Value-Based Care (VBC) is a healthcare delivery model that emphasizes improving patient outcomes while optimizing costs. It shifts the focus from the volume of services provided to the value delivered to patients. We hypothesize that innovative VBC intervention would be more cost-effective compared to standard care among individuals with poorly controlled hypertension.

**Methods:**

This study in Ghana will employ a mixed-methods evaluation design, a comprehensive and thorough approach that combines quantitative and qualitative methods. The quantitative component will involve a quasi-experimental study to measure the impact of the VBC intervention on quality of life, improved clinical outcomes, patient satisfaction, and enhanced financial protection among hypertensive patients registered with the National Health Insurance Authority. We will use a difference-in-difference analytic approach and a generalized estimation equation model with cluster-robust standard errors to quantify the impact of VBC, accounting for potential confounding variables. The qualitative component will involve in-depth interviews and focus group discussions to gather insights into the experiences and perceptions of the patients, caregivers, and policymakers involved in the VBC intervention and the benefits, barriers, costs of treatment, and challenges associated with the VBC intervention.

**Discussions:**

Despite the availability of safe treatment options for hypertension, most people with hypertension in LMICs do not have it controlled. There is currently a paucity of knowledge about the cost-effectiveness of VBC interventions in developing countries. This study aims to fill this knowledge gap and pave the way for more cost-effective hypertension treatment worldwide. The Ghana VBC intervention described in this paper is a pioneering approach to achieving safer, more consistent, and cost-effective care for hypertensive patients.

## Introduction

There has been a significant increase in the burden of non-communicable diseases (NCDs) in sub-Saharan Africa (SSA) over the past two decades, driven by demographic, socio-economic, and environmental factors such as unhealthy diets, reduced physical activity, hypertension, obesity, diabetes, dyslipidemia, and air pollution [1]. Hypertension is a major public health concern that contributes substantially to the rising global trend in morbidity and premature mortality [2]. It remains the leading cause of cardiovascular disease globally, and CVD remains the leading cause of death worldwide [3]. It is estimated that at least two-thirds of cardiovascular deaths now occur in low- and middle-income countries (LMICs), bringing a double burden of disease to poor and developing world economies [4,5]. Ferdinand et al., [6] showed that the highest levels of blood pressure worldwide have shifted from high‐income countries (HIC) to LMICs, and the World Health Organization (WHO) estimates that the prevalence of hypertension is highest in the African region, with about 46% of adults aged 25 years and older being hypertensive compared to 35% in the Americas and other HIC, and 40% elsewhere in the world [4,7]. An estimated 1.28 billion adults aged 30–79 years worldwide have hypertension, most (two-thirds) living in LMIC, and less than half of adults (42%) with hypertension are diagnosed and treated. It is estimated that approximately 1 in 5 adults (21%) with hypertension have this condition under control. Uncontrolled hypertension may increase the risk of stroke, myocardial infarction, cardiac failure, and renal failure, among others [8], and it accounts for approximately 9.4 million deaths annually [9]. It has been suggested that the prevalence of hypertension is increasing rapidly in SSA, and the estimated number of adults with hypertension in 2025 is predicted to increase to a total of 1.56 billion, with a disproportionate prevalence in developing countries, including sub-Saharan Africa, compared to high-income countries [10].

The prevalence of hypertension in Ghana from the medical literature varies. Some reported prevalence of hypertension ranges from 26-35%, with a higher prevalence recorded among men [11–13]. Others reported a higher prevalence of 59% [14], and it is estimated that two-thirds of the hypertensives were unaware of their condition, and approximately 50% of those with a history of hypertension on medication were controlled [14]. Additional studies report a very low prevalence of controlled hypertension among those who are on treatment in Ghana (approximately 3-12 %)[12,14]. Risk factors of hypertension in Ghana include but are not limited to age, marital status, gender, and residence (urban, rural), consumption of table salt, salted meat, alcohol, canned meats, smoking, and psychological factors, including stress and anxiety, higher education and socio-economic status, employment, access to medical insurance, overweight [15–17]. Other factors include poor adherence to medication, inadequate health system capacity for early diagnosis due to an increasing number of patients, inequitable distribution of healthcare facilities affecting access, financial sustainability of the National Health Insurance Scheme, and delays in reimbursement of claims to facilities that affect the health system’s ability to provide timely management of hypertension [18]. Healthcare facilities and practitioners’ use of non-standardized and uncalibrated blood pressure measuring equipment are among the challenges reported in the medical literature [18]. Low providers’ knowledge of hypertension and patients’ awareness, policy-level challenges precluded nurses from prescribing or dispensing antihypertensives and licensed chemical sellers from stocking same [19].

Despite the benefits of hypertension management in reducing cardiovascular morbidity and mortality, evidence from the medical literature shows that the burden of hypertension remains high and control rates are poor in LMIC [20,21] and the SDG goal of reducing cardiovascular disease mortality by 30% by 2030 may not be met [22]. There is evidence that most individuals are not adequately treated, and there is no value for money [3]. In particular, Health Insurance Schemes for healthcare today are based on rewarding volume, not value for the money spent [23] and VBC stipulates a healthcare reform and policy change that must focus on improving health and healthcare value for patients [24].

VBC is a healthcare delivery model that emphasizes improving patient outcomes while optimizing costs. It shifts the focus from the volume of services provided to the value delivered to patients. This value-driven form of reimbursement has emerged as an alternative to the traditional fee-for-service reimbursement, which pays providers based on the quantity of services delivered. In the standard care system, providers deliver care services in a fragmented manner. In a value-driven care system, experience and outcome measures drive care delivery. The costs of care are not calculated per care service but per outcome achieved. The VBC model revolves around health outcomes generated in the patients, which can be determined based on specific measures, such as the reduced need for hospital readmission, improvement in health-related quality of life, better patient satisfaction, and enhanced financial protection. These measurements are used to adapt and combine care services along a patient journey (in the form of bundles) to maximize the outcomes while minimizing the costs.

This study aims to implement and evaluate the impact of comprehensive value-based care (VBC) intervention on health-related quality of life, patients’ clinical outcomes, patient satisfaction, and financial protection among NHIA-insured hypertensive patients. In addition, the study will assess challenges associated with VBC intervention in providing quality and cost-effective hypertensive care at health facilities and the mitigation strategies needed to address these challenges.

### Study hypothesis

We hypothesized that a VBC approach to improving clinical outcomes (lowering blood pressure, improving blood sugar, body mass index, and waist circumference, improved health-related quality of life, reducing cost of care, increasing level of adherence to treatment, and reducing other risk factors), informed by detailed analysis of health system and patient-specific barriers, would be superior to standard care in individuals with poorly controlled or newly diagnosed hypertension. That is, we determined whether a VBC model that involves key stakeholders’ participation (patients, healthcare workers, NHIA, PharmAccess, and CHAG) via the provision of effective medications and a comprehensive treatment and management plan could substantially improve clinical outcomes and overall quality of life and simultaneously reduce the cost of treatment of hypertension and its complications.

### Intervention components

In a VBC model, healthcare providers are incentivized to deliver high-quality, cost-effective care and are held accountable for the outcomes achieved. The benefits of a value-based healthcare system extend to patients, providers, payers, suppliers, and society, and VBC has the promise to significantly increase the overall health gains of the population. To chart a different trajectory and build human-centered health systems that generate better long-term value for society, the National Health NHIA, in collaboration with PharmAccess and CHAG, will implement and evaluate a VBC intervention for hypertensive patients. This VBC intervention will include different aspects of VBC as outlined in the strategic framework for value-based care implementations [25]. This includes interventions that focus on understanding patient needs, patient-centered solutions such as behavioral and self-management interventions and integrating monitoring and learning systems for providers. Its aim is to align the interests of all stakeholders in the health system, including patients, providers, payers, and policymakers. VBC aligns these stakeholders with a common goal: achieving outcomes that matter to patients at the optimal cost. Each component of the intervention, such as the training of healthcare workers and providing strategies to improve adherence to hypertensive medication and clinical outcomes, will be implemented continuously for 12 months for the target populations (hypertensive patients and health service providers) with a contact period of approximately 2 hours every two weeks over 12 months. Engagements with project beneficiaries include face-to-face and virtual meetings. Other components of the intervention include understanding the shared health needs of hypertensive patients, designing tailored solutions to improve hypertensive outcomes, measuring health outcomes and cost, and the formation of patient groups to spearhead peer education on hypertensive management and designing more efficient ways to expand the partnership will be completed within 12 months.

### Content of the Intervention

- Training healthcare workers to improve their knowledge, attitude, and practices regarding hypertensive care. To assess the fidelity of implementation, we will utilize existing data collection instruments specifically designed to assess healthcare workers’ knowledge, attitude, and practices regarding hypertensive care. The tool will be administered before and after training of healthcare professionals.
- Provision of critical medical equipment and essential medicine lists to promote the quality of hypertensive care.
- Promote clinician-led patient follow-up.
- Provide training for hypertensive patients on strategies to improve adherence to hypertensive medication and clinical outcomes.
- Provide incentives to healthcare workers to deliver high-quality and cost-effective care.
- Utilize digital technology to improve behavioral counseling programs for hypertensive patients.
- Provide training for patients and caregivers on self-hypertensive management. A standardized data collection tool on self-hypertensive management will be developed and administered before and after training to assess the impact of the training programs.
- Form patient groups where they discuss and share knowledge on hypertensive management.
- Develop a value-based payment model.
- Implement an information technology program to track patients’ progress.

### Conceptual framework

**Fig 1:**
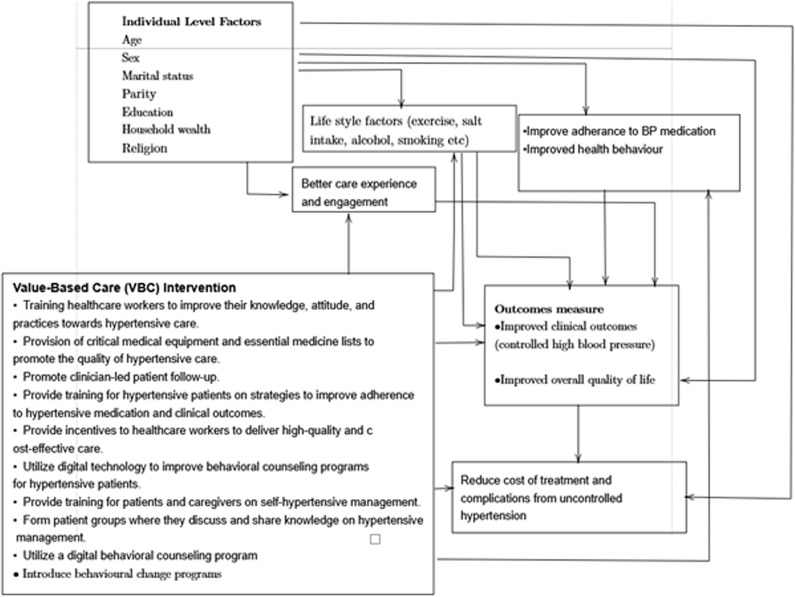
Framework underlying proposed hypothesis. Note: This framework was produced by the authors.

Fig 1 shows the framework for testing this hypothesis. This framework, developed by the authors, is based on subject knowledge of the VBC model and a literature review of factors associated with uncontrolled hypertension. The VBC interventions (such as the behavior change program, training of health professionals, providing incentives, etc.) are expected to better meet patient needs, improving patient care experience and health behaviors. These factors will contribute to better quality of life and clinical outcomes, preventing complications and reducing the overall costs for hypertensive patients in the VBC arm of this study. Our conceptual framework also follows the Reach, Effectiveness, Adoption, Implementation, and Maintenance (RE-AIM framework) [26] but with focus on the implementation and effectiveness of VBC.

### Expected outcome

The study will generate evidence regarding the effectiveness of VBC intervention on hypertension management in Ghana.

## Methods

### Evaluation design

The study will utilize a mixed-method evaluation design that incorporates both quasi-experimental design and qualitative design techniques to evaluate the impact of VBC on patients’ clinical outcomes, health-related quality of life, and financial protection among NHIA-insured hypertensive patients visiting CHAG facilities in Ghana. This study’s design is ideal compared to cluster-randomized control trials (RCT), even though RCT is more robust with minimal bias and high internal validity. However, we consider the randomization of facilities to receive VBC interventions untenable since facilities were pre-selected based on resource availability and the willingness of the facility in charge to implement VBC.

To improve the internal validity of the quasi-experimental design, control facilities that share similar characteristics with the intervention facilities will be carefully selected such that there will be no systematic difference between the intervention and control facilities, and changes in health outcomes could be attributed to the VBC. Each selected intervention facility will have a control facility in the same region and at the same administrative level of the health facility (hospital, health center, etc.) to minimize bias associated with the location and the level of health facility. In addition, the same inclusion criteria for hypertensive patients would apply in the intervention and control health facilities to minimize within-patient bias.

The VBC intervention will be implemented at the facility level, targeting healthcare providers and patients who are nested within health facilities and will be followed over time.

A baseline survey will be conducted in all the intervention and control facilities to assess the mean and prevalence of the outcome measures and their risk factors among hypertensive patients visiting these facilities on specialized clinic days. Hypertensive patients in the controlled health facilities will receive standard care as provided during the regular clinic days. To improve the control of hypertension and save costs, the intervention facilities will receive VBC intervention programs delivered by trained intervention managers. The intervention will be given for 24 months (2 years), but a midline survey will be conducted in 12 months and at 24 months post-baseline).

### Study settings

The study will be conducted in 32 Christian Health Association of Ghana (CHAG) accredited health facilities (hospitals and health centers) in five administrative regions of Ghana that provide services for hypertensive patients. Twenty health facilities will constitute the intervention facilities, and the remaining 12 facilities with similar characteristics to the intervention facilities will be selected within the same region as comparison health facilities to reduce the effect of selection bias that arises from quasi-experimental studies.

### Target population

This study will involve hypertensive patients who visit CHAG facilities in Ghana’s six administrative regions. It will also involve healthcare workers (hospital administrators, medical doctors, nurses, pharmacists, and laboratory technicians) and VBC implementers at PharmAccess, CHAG, and the National Health Insurance Authority (NHIA).

### Power analysis and sampling design

We assumed that if the value-based care is effective, both systolic and diastolic blood pressure will reduce by 5 mmHg on average among patients in the intervention facilities compared to patients in the control facilities [27]. We further assumed a power of 90% to detect a significant effect of the value-based care if it exists, type I error of 5%, attrition rate of 10%, standard deviation of 10 mmHg [27], average cluster size of 20 representing the average number of hypertensive patients seen on a clinic day per visit with a coefficient of variation of cluster sizes 0.5 and a design effect of 4.6 to adjust for correlation of the outcome measure at the facility level assuming an intra cluster correlation (ICC) of 0.15. We also assumed that the correlation between baseline measurements (or other predictive covariates) and hypertension is 0.5. The estimated number of health facilities will be 32 (16 control facilities and 16 intervention facilities). Ideally, 16 control health facilities should have been selected to increase the power of the study. Today, the intervention has already been started with stakeholder engagement and training, albeit at a slow pace and intensity in 20 health facilities. This implies that of the 32 facilities, the remaining 12 can serve as a comparison group, which corresponds to 712 hypertensive patients (450 patients in the intervention facilities and 262 patients in the control facilities). On average, about 20 hypertensive patients receiving care on clinic days who meet the inclusion criteria and consent to be part of the study will be interviewed per facility.

### Ethical considerations

The study has secured ethical approval from the Christian Health Association of Ghana (CHAG) Ethics Review Committee, with ethics approval number CHAG-IRB01012024. CHAG is the legally mandated institution required to provide ethical approval for all health-related research in CHAG-accredited health facilities. We emphasize that study participants will not be named in any report, and we will keep their identities confidential. Electronic data will be safeguarded in a password-protected folder, and only authorized users on the study team will be able to access it. The study will obtain written informed consent from all study participants.

### Informed consent

The study obtained written informed consent from all study participants, which included statements on the study objectives, possible risks, benefits, compensation, privacy, and confidentiality.

### Study participant’s inclusion criteria

Eligible health facilities will be CHAG-accredited health facilities providing hypertensive services for patients on clinic days. Eligible participants will comprise all consenting patients (male and female), age 18 years and above, visiting CHAG facilities on a clinic day because of past or current diagnosis of hypertension. That is, all patients visiting hypertensive clinics with the following:

1) Systolic blood pressure (SBP) ≥ 160 mm Hg is recorded at the first onsite screening.
2) SBP 140-159 mm Hg is recorded at first onsite screening, AND the participant reported a medical diagnosis of hypertension or is taking anti-hypertension medication.
3) SBP less than 140mm Hg is recorded at first onsite screening, AND the participant reported a medical diagnosis of hypertension or is taking anti-hypertension medication at the health facility.
4) Participants did not meet criteria 1-3, but SBP 140-159 mm Hg was recorded on two separate screenings at the facility within 1 hour.

### Exclusion criteria

Participants will be excluded if they meet these criteria but are unable to participate in the study due to sickness or any other form of serious illness that makes it impossible for him/her to grant an interview, refuse to consent, concurrently participate in any other intervention-based study that would compromise the protocol of VBC, had a severe comorbid condition with life expectancy < 1 year, or factors likely to interfere with study participation or with their ability to complete the study.

### Dropout

Participants will be considered dropouts if they are unwilling to continue their participation for any reason, withdraw due to health problems, or if someone dies within the study period. To minimize the rate of loss to follow-up during our subsequent interviews, we will select hypertensive patients who are likely to visit the same health facility for hypertensive care for the next 12 months. There will be regular follow-up reminders and incentives for continued participation.

### Collection of qualitative information

A nested qualitative study will be conducted within the quasi-experimental design. Semi-structured in-depth interviews of key informants will be conducted to capture the perspectives and experiences of the value-based care intervention and perceived barriers and enablers faced during the implementation of the intervention, the impact of the VBC on the quality of life of hypertensive patients, etc. We will develop an interview guide based on the study objectives and previous literature to align with the research goals. The qualitative part of the study will be conducted alongside program implementation to identify challenges and barriers associated with VBC implementation and to identify strategies to improve program adaptation.

Using a purposive sampling approach, data will be collected from all targeted populations, including patients, service providers (clinicians, nurses, pharmacists, laboratory technicians), PharmAccess, CHAG, NHIA, and all other relevant stakeholders. At least 30 in-depth interviews with key informants will be conducted (15 interviews in the intervention facilities and 15 interviews in control facilities). The qualitative interviews will be truncated when we reach thematic saturation.

In addition, a total of three key informants will be interviewed from NHIA, CHAG, and PharmAccess. All interviews will be digitally recorded and transcribed verbatim after translating from the local language to English. Transcripts will be reviewed through a quality assurance system for accuracy and consistency. Transcripts that are found to meet the quality standards after a thorough review will be analyzed using a thematic content analysis approach. A sample of each set of transcripts from the different targeted populations will be reviewed by the consultants. Themes based on the interview guide underpinned by the objectives will be developed with consultants coming to a consensus on where differences exist in themes.

### Qualitative analysis

Researchers will independently code the transcripts by assigning codes to segments of text that capture key concepts and ideas. Codes will be refined and grouped into broader themes that represent the core findings of the interviews. Data will be analyzed through the framework approach [28], which embraces inductive (emerging codes) and deductive (a priori code from the utilization-focused evaluation module) methods. The framework approach involves six (6) iterative steps: (1) familiarization with the data, (2) generating initial codes or assignment of primary codes to the dataset to describe the content, (3) searching for patterns and codes across the different interviews, (4) codes revision, (5) codes definition and (6) producing the report through the interpretation of results. The inductive method will involve steps 1 and 2, while the deductive method will involve steps 3 to 6. The codes will be discussed and compiled into a codebook to guide the coding and categorization of the data. The deductive analysis approach will ensure coding data units according to evidence relating to impact, challenges, and mitigation strategies associated with implementing VBC at the health facilities. The coding process will be done in QRS NVivo version 12 to enhance the rigor and audit trail of all data coded.

### Training of enumerators and supervisors

Training of field data collectors, piloting of data collection instruments, the actual field data collection, data cleaning, analysis, and report writing will start in February 2025 and end in August 2025.

The training will focus on understanding the content of the data collection instrument, key objectives of the VBC project, data collection methods, ethical consideration in scientific research, code of conduct during field surveys, informed consent, communication channel, ensuring data quality, collection of GPS coordinates of the health facilities and the households, conducting interviews, checking completed questionnaires, digital data entry, and ensuring standard operating procedures are followed. Specifically, the training will involve an overview of the activity and evaluation methodology, informed consent and confidentiality, data quality assurance procedures and quality checklists, mock practices with each tool, qualitative data collection practices (facilitation, inclusiveness, probing), piloting the tool, securing data storage and transfer, common troubleshooting and daily reporting and oversight. Training will incorporate a complete review of the survey instruments and role-play to simulate real-life interview scenarios. Supervisors will also receive special leadership training skills during this period to efficiently coordinate the fieldwork activities and ensure adherence to the project timelines.

### Data integration

Quantitative and qualitative findings will be triangulated to better understand the impact of the VBC intervention, challenges, and mitigation strategies associated with its implementation at the health facilities. Qualitative data will enrich the quantitative findings by providing an in-depth understanding of hypertensive patients’ and other stakeholders’ lived experiences regarding the VBC intervention. The qualitative data will provide an understanding of how VBC impacts patient health outcomes, minimize cost of treatment, improved quality of care, enhanced financial protection, and identify bottlenecks in the implementation of VBC intervention to achieve improved health outcomes among hypertensive patients.

With the analysis and integration of data from two sources obtained by the mixed methods approach, the findings can be corroborated, and the weakness or bias of any of the methods or data sources can be compensated for by the strengths of another, thereby increasing the validity and reliability of the results.

### Primary outcome measures

The primary outcome measures include systolic blood pressure and diastolic blood pressure. Participants will be considered to have high blood pressure if recorded SBP ≥ 130 mmHg and/or DBP ≥ 80 mmHg [29]). These primary outcomes will be compared between intervention and control groups after 12 and 24 months of implementing the value-based care intervention. We will also compare the difference in mean systolic and diastolic blood pressure levels at baseline, midline, and post-intervention periods for the control and intervention groups. Blood pressure will be measured using a calibrated Omron Premium Blood Pressure Monitor Device, Omron HEM-7322 (Australia). Blood pressure will be measured from the right arm with the person sitting upright after 3 min of rest, and a further measurement will be taken following a period of at least 3 minutes of rest. If a major difference is observed between the first two measures, a third measure will be taken, and the nearest two measures will be recorded. The two readings will be averaged separately for SBP and diastolic blood pressure (DBP). We will follow the 2017 American College of Cardiology and American Heart Association to measure blood pressure [30].

### Secondary outcome measures

The secondary outcome measures include blood sugar level, health-related quality of life, weight and height, waist, hip circumference and waist-to-hip ratio (WHR), adherence to medication, health professional knowledge, depression anxiety, stress, and the average cost of care.

#### Blood sugar.

The blood sugar level will be monitored using the HbA1c test. A normal HbA1c level is below 5.7%, 5.7% to 6.4% indicates prediabetes and 6.5% or more indicates diabetes based on the National Glycohemoglobin Standardization program [31]. Within the 5.7% to 6.4% prediabetes range, the higher your HbA1c, the greater your risk of developing type 2 diabetes.

#### Health-related quality of life.

The EQ-5D will be used to assess the health-related quality of life of the study participants [32]. It consists of five sets of questions measuring five dimensions: mobility, self-care, usual activities, pain/discomfort, and anxiety/depression. Each question is rated on a 5-point scale (1 – No problem to 5 - Unable/extreme problems). Using a value set, the individual health rating is converted to a health-rated quality of life index ranging from 0 (dead) to 1(fully healthy). The value set is a set of weights applied to each severity level in the 5 dimensions. Higher weight is applied to high severity while low weight is applied to low severity on quality of life.

#### Weight and height.

This study will use the portable stadiometer (SECA, United Kingdom) with an accuracy of 1 mm to measure the heights of the participants, i.e., from the heel to the highest point of the head, will be measured. The study participants will be asked to take off their shoes during the measurement. The weight of the participants will be measured using a calibrated weighing scale to the nearest 0.1 kg. Then, the Body Mass Index (BMI) for each participant will be calculated as follows: $weight/height^{2}$.

#### Waist, hip circumference, and WHR.

The waist circumference of each participant will be measured using a flexible tape to the nearest 0.1 mm. The point of measurement, i.e., above the hip bone, will be fixed for all respondents, and the respondents will be advised to relax the stomach by breathing out gently and not to suck in the tummy. The tape will be placed properly and parallel to the floor without compressing the skin. The measurement will be taken around the waistline at the end of normal expiration. The area with maximum protrusion of the buttocks on standing with the feet close together will be considered as the reference point for the hip circumference measurement. We will estimate the waist-to-hip ratio (WHR) from the values obtained from the two indicators.

#### Compliance to hypertensive medication.

The Hill-Bone High Blood Pressure Compliance Scale [33] will be used to assess the level of compliance across three behavioral domains critical for HBP care and control: 1) reducing sodium intake, 2) appointment keeping, and 3) medication taking. The scale has 14 items rated on a four-point scale from 1 – Never to 4 - All the time. The total score is computed by summing the response ratings, and the score ranges from 14 to 46. A high score is indicative of noncompliance, and a low score is indicative of compliance.

#### Health professional knowledge.

A newly validated international questionnaire will be used to assess health professionals’ knowledge of hypertension diagnosis [34]. The tool enables objective and comprehensive assessment of medical and nursing professionals’ knowledge of the initial diagnosis of hypertension. The tool has 23 items and assign equal weight for each of the items.

#### Depression, anxiety, and stress.

Depression, Anxiety, and Stress Scale (DASS-21) will be used to assess depression, anxiety, and stress among hypertensive patients. The DASS-21 is a self-report measure in which hypertensive patients will rate the frequency and severity of experiencing negative emotions over the previous week. Frequency/severity ratings are made on a series of 4-point scales (0 =  did not apply to me at all, 3 =  applied to me very much, or most of the time).

#### Cost of treatment.

This study will use structured questionnaires to measure out-of-pocket health expenditures (direct and indirect cost) before and after VBC intervention among patients in the intervention and control health facilities.

#### Cost of implementing VBC intervention.

Intervention costs for VBC in this study refer to the resources used to operate and deliver the intervention. The intervention cost of the VBC hypertension management program will include the cost of training for medical practitioners to deliver lifestyle coaching sessions, the cost of the venue for training, the cost of delivering lifestyle coaching sessions, the cost of medication, the cost of communication for providers to reach out to patients to improve adherence, cost of periodic reviews of outcomes and costs leading to continuous improvement cost of implementing and testing digital behavioral counseling program, patient tracking, and cost of training patients on self-management. Overall, the ingredients approach will be used to collect all relevant cost data. This approach means that all relevant materials and items required for implementing each activity will be quantified and valued. For example, training of healthcare providers, patients, etc. Personnel costs, including per diem for trainees, honorarium for trainers, venue, stationery, and other relevant logistics, will be costed to measure financial cost. Where relevant capital items will be costed and annualized at a 3% discount rate. Financial costs will be estimated by calculating direct medical (e.g., drugs, laboratory tests) and non-medical costs (e.g., transportation, personnel costs, etc). Indirect cost will also be measured for productivity losses to appreciate the opportunity cost of the intervention. This will include travel time, time spent in training, etc. The total economic cost is the sum of the direct and indirect costs. The study will use the health care provider perspective, payer (NHIS) perspective and societal perspective. If proven effective, the consequences of implementing this intervention include increased quality of life from patients achieving blood pressure control and savings in resources by avoiding hospitalizations that could have otherwise occurred. The following steps will be taken to micro-cost the VBC intervention.

Step 1: This will involve defining the VBC processes and identifying the types of resources that are used in carrying out those processes. This step could include reviewing documents or literature, conducting a site visit, or talking to core staff. The purpose is to gain a detailed understanding of how the VBC operates and to identify an inventory list of what resources are involved and how they are used.

Step 2: We will systematically measure the unit quantity of each type of resource consumed. That is, we will determine the quantity used for each type of resource identified in step 1.

Step 3: Each type of resource will be assigned a unit cost, and for each resource type, the unit cost will be multiplied by the unit quantity and aggregated to find the total cost.

Step 4: Finally, adjustments will be made to any assumptions, such as the value of a certain resource or assumptions that might influence the estimated quantity of resources consumed as part of the implementation of the VBC intervention, to test the robustness of the estimates and show how it might change under different sets of assumptions.

### Selection of comparison facilities

Comparison health facilities were carefully selected to reflect the characteristics of the intervention facilities. For each intervention facility, a control facility will be selected from the same region and the same level of care as there are marked differences in the availability of qualified medical personnel, logistics (medicines and supply), and poverty across the regions. Patients in the controlled facilities will only receive the usual standard hypertensive care, and they will not receive the VBC-based intervention. Standard hypertensive care in this study refers to the usual care they receive when they visit hypertensive clinics.

### Potential confounders

The independent variables of interest in this study include the respondent’s sex, age in years, current marital status, educational level, religion, employment status, religious affiliation, valid NHIS card, study location, household size, lifestyle factors (exercise, anxiety, depression, stress and diet, alcohol and smoking), and household wealth.

### Data management

Each participant will be assigned a unique identification number after the baseline survey. This will facilitate and reduce the errors in linking lab-based clinical outcomes to other patients’ records. The research team members will develop the study tool for the baseline, midline, and endline surveys. Data collection would be done electronically during face-to-face interviews at the facility or respondent homes using Open Data Kit. Prior to the commencement of fieldwork, there will be recruitment for the positions of laboratory scientist for each health facility, field data collectors (enumerators), and supervisors. The laboratory scientist will lead the collection of all blood samples for testing. A one-week training would be organized for enumerators and field supervisors focusing more on the questionnaire’s definitions of key outcome variables of interest and how to ensure data quality. The training of enumerators and supervisors will be preceded by training on the ethics of research involving human subjects. Training will incorporate a complete review of the survey instruments and role-play to simulate real-life interview scenarios. Supervisors will also receive special leadership training skills in order to efficiently coordinate the activities of the fieldwork and to ensure adherence to the timelines of the project. All interviews would be completed by small teams of enumerators led by a field supervisor. The supervisors would be responsible for monitoring the field enumerators. The enumerators, team leaders, and field supervisors would be selected based on their experience conducting longitudinal cluster randomized studies and fluency in the relevant local languages. To reduce the unit non-response rate, each enumerator would approach a household up to three times to locate respondents. In addition, the telephone number of the respondent and close relative will be obtained after informed consent to follow up with patients throughout the study. The training manual will be used throughout the training period and as a reference throughout the survey. Two days will be allocated for the pre-test of questionnaires and discussions with supervisors and enumerators to ensure familiarity and address outstanding issues arising from the pre-test at Ghana Health Service (GHS)try facilities. The study investigators will be responsible for data quality control issues handled on three different levels. The first level is the real-time logic and range checking built into the online data entry system. The second level of quality control involves the study investigators conducting real-time daily checks. Checks will identify complicated and less common errors. The third level of quality control involves local monitoring, where data in our database will be checked, and if errors are identified, research assistants who commit these errors will be identified using the system and asked to correct them before moving to the next stage of data collection.

### Ethics approval and consent to participate

The study has secured ethical approval from the Christian Health Association of Ghana (CHAG) Ethics Review Committee, with ethics approval number CHAG-IRB01012024. CHAG is the legally mandated institution required to provide ethical approval for all health-related research in CHAG-accredited health facilities. The study will be conducted in accordance with the ethics guidelines of the Declaration of Helsinki. Informed consent will be secured from each study participant. The participants’ confidentiality will be assured by not including names or personal identifiers during data collection, analysis, and reporting and respecting the privacy of study participants during interviews. Participants’ right to refuse participation, not answer any questions they don’t want to, or withdraw participation after enrolling will be fully respected. Regarding qualitative interviews, confidentiality will be maintained throughout the interview and during audio recording and transcription. Completed data collection tools, including audio recordings, will be password protected, anonymized, and coded to ensure privacy; consent forms will be stored separately to ensure that identifying information cannot be linked. All electronic data will be encrypted to prevent accidental access to data in which a respondent’s identity may be revealed from the context description.

### Statistical analysis

Analysis and reporting of the effect of the VBC will follow the Consolidated Standards of Reporting Trials guidelines for cluster randomized controlled trials [35]. The study will utilize descriptive and inferential statistics to address the study objectives. Mean, standard deviations, minimum and maximum observations, and confidence intervals will be reported for normally distributed continuous outcome measures. The median and interquartile range will be reported for non-normally continuous variables. Both graphical (histogram superimposed with a normal distribution curve) and numerical methods (Shapiro Francia and skewness and kurtosis test) will be used to determine whether continuous variables approximate normality. Categorical variables will be summarized using frequencies and percentages of each category, and the association between categorical variables will be analyzed using the chi-squared test of independence (with Yates correction for 2 ×  2 tables) or Fisher’s exact test in cases where expected frequencies are low. The study will employ both parametric and non-parametric inferential statistical methods. The student’s t-test (for parametric variables) or the Wilcoxon Rank sum test (for non-parametric variables) will be used to test the hypothesis of difference in means or medians of continuous outcomes between intervention and control groups. Z-test for comparing the differences in proportion will be used to test the hypothesis of the difference in the proportion of binary outcome measures between intervention and control. To quantify the impact of the VBC intervention on the outcome measures of interest, intention-to-treat-based difference-in-difference (DID) analysis with a multivariable ordinary least square regression, quantile regression, binary logistic regression model, Negative binomial and Poisson regression models with robust standard errors will be used depending on the measurement scale of the outcome measure. The analysis of both primary and secondary outcome measures will account for the clustering effect and use a mixed effects model considering the facility as a random intercept effect. Additionally, individual-level marginal modeling (population-averaged model) using generalized estimating equations (GEEs) with an exchangeable correlation structure will be used. The consistency of VBC’s impact on the primary outcome will be explored in predefined subgroups, including sex, age categories, education, region, marital status, and comorbidities. We will test whether the effects varied by subgroups using tests of interaction-based models between subgroup variables and the treatment group. In addition, sensitivity analysis will be performed using DID model that integrates propensity score matching techniques to reduce confounding bias associated with non-experimental study designs.

All statistical analyses will be conducted using Stata MP version 17 (StataCorp. 2021. Stata Statistical Software: Release 17. College Station, TX: StataCorp LLC), and a two-sided test at the 0·05 level will be considered to be statistically significant. Final results will be expressed as mean/proportion (95% CI) in each group, and two-sided p values will be reported.

## Discussion

The Ghanaian National Health Insurance Scheme (NHIS) rewards the volume of services provided to hypertensive patients, not the value for the money spent. We developed a research protocol to assess the cost-effectiveness of value-based care intervention for hypertensive patients. This protocol will generate evidence to shape public opinion regarding the effectiveness of value-based care on hypertension management and the possibility of policy formulation to scale up to other health facilities and eventually incorporate it into health insurance.

Despite the benefits of hypertension management and the availability of safe and low-cost medications in reducing cardiovascular morbidity and mortality, evidence from medical literature shows that the burden of hypertension remains high, and control rates are poor in developing countries. Controlled hypertension is one of the priority areas identified by the WHF for action if the target of a 25% reduction in premature CVD mortality by 2025 is to be achieved [36].

This study aims to implement and evaluate an innovative VBC intervention in Ghana. Our study will assess the effectiveness of VBC on health-related quality of life, clinical outcomes, and cost of treatment. The VBC approach to healthcare allows health professionals to think differently about their role within the larger care team and the services they provide in general. For instance, in the absence of value-based care, most physicians are largely concerned with what happens in the consulting room and, in most cases, do not follow up with patients outside office hours to enquire about progress made after the first appointment.

Our proposed VBC intervention starts with identifying and understanding hypertensive patients whose health and related circumstances create consistent needs relative to improved health outcomes and cost. To achieve this, the study plans to involve an interdisciplinary team of caregivers, policymakers, and patients to design and deliver comprehensive solutions to health-related challenges associated with providing quality care at an affordable cost. The research team will measure the health outcomes and costs of care for each hypertensive patient during baseline, midline, and endline data collection and use the information to drive ongoing improvements, identify challenges and mitigation strategies, and assess the impact of VBC on the selected outcome measures. VBC interventions have been shown elsewhere to improve the quality of care, lower rates for some measures of acute care utilization, and lower actual payments received by the delivery system [37]. Others have argued about the need to introduce regulations to end coverage and price discrimination based on health risks or existing health problems and measure and report their subscribers’ health outcomes, starting with a group of important medical conditions [38]. However, we need to provide evidence of the effectiveness of VBC interventions in improving the quality of care and reducing the cost of treatment to justify the need to shift focus to value-based healthcare. The health system is confronted with issues regarding the quality and cost of care, and the current payment system that encourages volume-driven care rather than value-driven care is a contributing factor [23]. Healthcare providers and facilities gain increased profits by delivering more services to more people, resulting in increased healthcare costs without corresponding improvement in health outcomes. VBC could mitigate the impact of factors that drive healthcare cost of hypertensive treatments, such as the prevalence of hypertension in the population (for example, how many people have hypertension), the number of times patient receives care they require per condition (for example, how many hypertension-related comorbidities a person with hypertension has); the number and types of health care services the hypertensive patient receives in each episode (for example, when a person has uncontrolled hypertension, does he or she receive heart-related treatment induced by hypertension or simply medical management?); the number and types of processes, devices, and drugs involved in each hypertensive service and finally, the prices of each of those individual processes, devices, and drugs. There are some limitations of the study including social desirability biases generally associated with non-observational studies and self-reported bias of two of the outcome measures (health-related quality of life and medication adherence) which could influence the outcome measures of interest.

To the best of our knowledge, no studies have been conducted in Ghana and, to a large extent, LMIC to quantify the impact of value-based care on health-related quality of life, improved patient clinical outcomes, patient satisfaction, and enhanced financial protection among NHIA-insured hypertensive patients. This evaluation study will (1) allow us to generate evidence on the impact of VBC on the aforementioned outcome measures, identify challenges and mitigation strategies associated with the implementation of VBC interventions, (2) help to sensitize the policymakers on the need to shift from volume-driven care to value-driven care, and finally (3) allow a debate among health providers, policymaker, Civil Society Organizations and all other relevant stakeholders on the need to expand VBC to other facilities assuming the intervention prove to be cost-effective.
